# Establishment of a Percutaneous Coronary Intervention Registry in Vietnam: Rationale and Methodology

**DOI:** 10.5334/gh.782

**Published:** 2020-04-08

**Authors:** Hoa T. T. Vu, Hoai T. T. Nguyen, Hung M. Pham, Loi D. Do, Quang N. Nguyen, Richard Norman, Rachel R. Huxley, Ngoc M. Pham, Crystal M. Y. Lee, Christopher M. Reid

**Affiliations:** 1 School of Public Health, Curtin University, Perth, AU; 2 Thai Nguyen University of Medicine and Pharmacy, Thai Nguyen, VN; 3 Vietnam National Heart Institute, Hanoi, VN; 4 College of Science, Health and Engineering, La Trobe University, Melbourne, AU; 5 The George Institute for Global Health, University of New South Wales, Sydney, AU; 6 Menzies Institute for Medical Research, University of Tasmania, Hobart, AU; 7 School of Psychology and Public Health, La Trobe University, Melbourne, AU; 8 Boden Institute of Obesity, Nutrition, Exercise & Eating Disorders, University of Sydney, Sydney, AU

**Keywords:** methodology, percutaneous coronary intervention, registry, Vietnam

## Abstract

**Background:**

In lower- and middle-income countries across Asia there has been a rapid expansion and uptake of percutaneous coronary intervention (PCI). However, there has been limited routine collection of related data, particularly around quality, safety and cost. The aim of this study was to assess the viability of implementing routine collection of PCI data in a registry at a leading hospital in Hanoi, Vietnam.

**Method:**

A Vietnamese data collection form and collection strategy were developed in collaboration with the Vietnam National Heart Institute. Information on patient characteristics, treatments, and outcomes was collected through direct interviews using a standardised form and medical record abstraction, while PCI data was read and coded into paper forms by interventional cardiologists. Viability of the registry was determined by four main factors: 1) being able to collect a representative sample; 2) quality of data obtained; 3) costs and time taken for data collection by hospital staff; and 4) level of support from key stakeholders in the institute.

**Results:**

Between September 2017 and May 2018, 1,022 patients undergoing PCI were recruited from a total of 1,041 procedures conducted during that time frame. The estimated mean time to collect information from patients before discharge was 60 minutes. Of the collected data fields, 98% were successfully completed. Most hospital staff surveyed indicated support for the continuation of the activity following the implementation of the pilot study.

**Conclusions:**

The proposed methodology for establishing a PCI registry in a large hospital in Vietnam produced high quality data and was considered worthwhile by hospital staff. The model has the potential opportunity for replication in other cardiac catheterisation sites, leading to a national PCI registry in Vietnam.

Coronary heart disease (CHD) is consistently the leading cause of death worldwide, responsible for approximately 16.6% of total deaths in 2016 and places a large economic burden on the population [1,2]. Since its inception in 1977, percutaneous coronary intervention (PCI) has been recognised as a valuable procedure for treating CHD patients and has become a common part of routine practice worldwide [3,4]. The Asia-Pacific region is home to nearly 60% of the world’s population, where CHD is now a leading cause of mortality [5,6] and the development of PCI registries is of growing interest [7,8,9]. As a clinical quality registry, a PCI database is an important mechanism for monitoring and benchmarking the performance of clinical care, improving safety and outcomes, contributing to reducing treatment cost and regulating guidelines [10,11]. Nonetheless, there remains wide geographic variation in terms of the organisation, operation, management, sustainability and utilisation of data collected of PCI registries in Asia. Additionally, data are limited regarding the participation of less economically developed countries, particularly those in the South-East Asia region, including Vietnam [9,12,13,14].

As a nation undergoing rapid economic and epidemiological transition, Vietnam has experienced a high burden of CHD, causing more than 58,000 deaths (11.6% of all mortality) in 2017 [15]. Vietnam began to adopt PCI in 1995 at the Vietnam National Heart Institute (VNHI), and has to date introduced this procedure to approximately 70 cardiac centres nationwide [16]. The annual number of PCI procedures is relatively large and increasing; for instance, there were 2,250 patients receiving this technique in 2013 in a single national centre and there is a 15% increase annually [17]. Notwithstanding its widespread use, there has been no PCI registry in Vietnam.

This paper presents the rationale, design and conduct of a pilot PCI registry model in Vietnam. The viability of implementing routine collection of PCI data was also assessed and documented. If viable, this would be the initial step in developing a model for an expanded PCI registry in Vietnam. The major objectives of this pilot study are 1) to describe the implementation experience at a large cardiac centre in Vietnam; 2) to describe the methodology for developing the PCI registry in Vietnam; and 3) to report on the viability of the strategy as a model for the nation.

## Methods

### Study setting

This PCI pilot registry project was conducted at the VNHI. Located in Hanoi, the capital city of Vietnam, VNHI is the biggest cardiac and referral centre in the country, providing the highest quality of healthcare services for cardiovascular patients in the country. Numerous advanced catheter-based therapies including complex coronary stenting, aortic stent grafting, transcatheter cardiac structural interventions, have become integrated into routine clinical practice at VNHI. As a 450-bed medical institution, VNHI receives around 17,000 inpatients and 80,000 out-patients annually, from across Northern Vietnamese provinces. The number of cardiac interventional procedures undertaken at VNHI is increasing from approximately 2,800 in 2004 to 12,000 in 2018 [17]. In addition, VNHI is an education and training centre, from which cardiovascular technologies and therapies are transferred into practice at other lower level medical institutions. VNHI was therefore selected for implementing the pilot PCI registry to reflect the contemporary practice of PCI in Vietnam.

### Establishment of dataset

This study adapted the current versions of standardised data abstraction forms developed for the Victorian Cardiac Outcomes Registry (VCOR), Australia [18], including the standard case report form (CRF) and dataset definitions for all fields. The state-wide VCOR was built on the Melbourne Interventional Group registry [19], in which PCI data elements are in line with a number of current interventional registries worldwide, for instance, the American College of Cardiology – National Cardiovascular Data Registry [20]. The standard dataset aimed to collect the minimum standard data and avoid cumbersome management:

1. The three page baseline survey, administered at time of presentation for procedures, contains 13 sections: patient details, admission data, clinical symptoms, clinical presentation, pre-procedural left ventricular function, risk factors, renal status, medication, procedure details, post-procedural cardiac biomarkers, in-hospital complications, discharge details and medications.
2. The one page survey for 30-day and 12-month follow-up, including four sections: patient details, outcomes, medications and quality of life at 30 days and 12 months.

The VCOR data collection forms were translated into Vietnamese, and revised by two Vietnamese clinical cardiologists to reflect local practice. After discussion, consensus was reached around the addition of some new elements into the Vietnamese data collection forms, such as patient details (e.g. medical record number, ethnic group, poverty status, educational level, occupation, and income) and risk factors (e.g. smoking, dyslipidaemia).

The Vietnamese data collection forms were designed in the TELEFORM software [21] and printed in paper records. The specific questions for all three data collection points are presented in the Appendix.

### Data collection

Following the finalisation of the data collection form, data recruitment commenced in September 2017. Potential participants were patients who underwent PCI at VNHI during the study period and met the following criteria: (1) Vietnamese residents aged 18 years and over; (2) Had at least one active phone contact number; and (3) Able to communicate, understand the information sheet and did not opt-out of future follow-ups by the time of discharge. There were no exclusion criteria. Under the strict clinical audit and strategy for data collection, baseline data collection was conducted over a 9 month period (from September 2017 to May 2018), followed by the 30-day follow-up, while the 12-month follow-up is underway.

### Baseline survey

Baseline data were collected using a paper-based form through interviewing patients, visiting the catheterization laboratory where PCI was performed and extracting information from medical records. The data manager at VNHI and research assistants were responsible for conducting these activities. Compliance with the project protocol was supervised by staff trained in clinical audit processes. The patient interviews were largely conducted in wards, following the index PCI and prior to discharge when patients were medically well enough, as assessed by the responsible physician. Data on the index PCI (e.g. the PCI indication, entry location, adjunctive devices, lesion characteristics, in-stent restenosis, stent thrombosis, and stents used) were obtained from the catheterization laboratory. Images of coronary lesions were stored on protected disks, and printed, read and coded by a cardiologist. Other information was abstracted from medical records including time of admission, in-hospital management, medications used, clinical tests and pre-discharge complications (e.g. renal impairment, cardiogenic shock, bleeding [classified by the Bleeding Academic Research Consortium (BARC)]) [22], stroke, new or current myocardial infarction, target vessel or lesion revascularisation after the procedure (PCI or coronary artery bypass grafting). It took approximately one hour to complete a CRF on average.

### Follow-up surveys

The 30-day and 12-month follow-up surveys were designed to capture data on the combined endpoint of major adverse cardiac and/or cerebrovascular events such as all cases of death, new or recurrent myocardial infarction or stent thrombosis, target vessel revascularisation or stroke; bleeding (BARC) [22]; rehospitalisation; medication use and health quality of life (mobility, personal care, usual activities, pain/discomfort, anxiety/depression, and own health state today) of participants after the index PCI.

At 30 days, a face-to-face interview was conducted by the data manager or research assistants if the participant was physically present at VNHI; otherwise, a phone interview was used. Supplementary information from a heart ultrasound and blood tests was recorded during the face-to-face interviews at VNHI, which lasted approximately 30 minutes.

The 12-month follow-up survey is beyond the scope of the current report, however the planned methods are as follows. A 15-minute phone interview is being undertaken by trained research assistants to obtain information from the patients directly. First-degree relatives are used as the proxy if the participant is not contactable. At least three attempts will be made to contact the participants. If patients have additional concerns regarding their health, then consultation will be available upon request following the interview.

### Perspectives from VNHI

We also conducted an online survey with qualitative open-ended questions to explore perspectives on the implementation of the registry and identify key factors associated with successful implementation of this new model at VNHI. We approached all clinical, nursing and leadership staff involved in coronary interventional activity. The survey that contained 10 questions was administered using Qualtrics Research Suite (Qualtrics, Provo, UT), a web-based tool that allows researchers to build, distribute, and analyse online surveys in real time. Analysis of qualitative data was guided by the principles of the conventional and summative content analysis [23]. Briefly, responses obtained from participants were coded to identify and categorise different themes together with performing word counts. The interpretation focused on the several key factors that may influence the development and implementation of PCI registry in Vietnam.

### Registry viability

This is the first study focusing on developing a model to collect data on the contemporary practice of PCI at the largest cardiac institute in Vietnam. The viability of the pilot PCI registry as a model for a national registry in Vietnam was determined by the following elements:

- Being able to recruit a representative sample into the registry.
- The quality of data collected, determined by data completeness and audit activities.
- Costs and time taken to collect the data by hospital staff.
- The level of support for the activity from patients, clinical staff and the cardiac institute.

### Ethics approval

Ethics for the study was approved by the Curtin University Human Research Ethics Committee (HRE 2017-0378). Every participant was provided with a Patient Information Sheet in which the purpose of the study, activities and rights of participants were described clearly. Participating in this study was voluntary and participants had the right to decline their participation or withdraw from the study at any time without any consequence via an ‘opt-out’ consent. A unique ID was assigned to each participant and linkable to the private information such as name, age, address, and phone numbers for follow- ups. All information that identifies participants was coded and stored confidentially.

## Results

### Implementation experience

**Figure 1:**
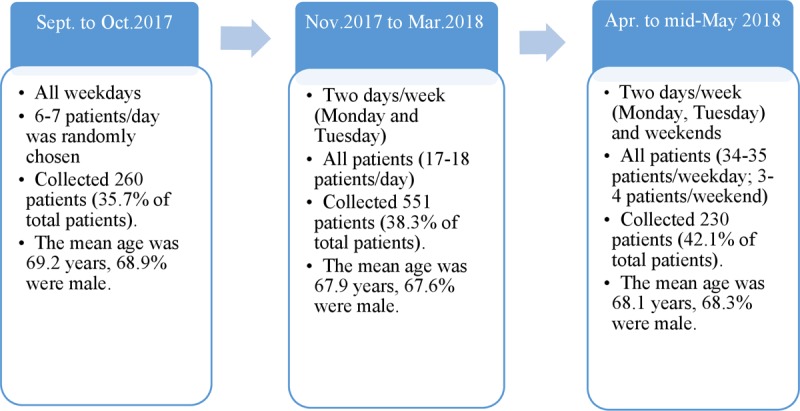
The data collection strategies.

During the study period (from September 2017 to May 2018), patients who underwent PCI at VNHI and met the inclusion criteria were approached and invited to take part in the study. Three strategies were used to enrol patients as the pilot study progressed, with changes applied to recruitment and the corresponding results (Figure 1). The modification of the data collection strategy was implemented to ensure that, in the absence of resources to capture all cases, we were able to capture a representative sample and minimise the potential for selection bias in registry enrolment when a single data manager was responsible.

For the first period of data collection (September and October 2017), 6–7 patients were randomly selected from the total number admitted of each day. However, to eliminate the potential bias of choosing only good prognosis patients (i.e., without complications prior to and after the procedure), we moved to second approach (from November 2017 to March 2018) which aimed to collect all cases in two weekdays. Two particular weekdays (Monday and Tuesday) were chosen to capture data from all operators including two teams of interventionists who performed PCI at VNHI on alternate days during the week. Notably, this second period of data collection coincided with Tet (Lunar New Year’s holiday), the biggest cultural event in the year, which may explain a reduction in the number of patient visits. The actual number of patients recruited in the period one and three may be generalizable to the rest of the year (i.e., from June to August) because there were no similar events in the year. Finally, the last period (from April to mid-May 2018) included all patients undergoing PCI on two weekdays and the weekend, which was designed to capture both the normal practice (weekdays) and acute cases (weekend).

A total of 1,028 eligible coronary patients were approached and invited from a total of approximately 2,800 patients undergoing PCI at VNHI during the study period. Six patients refused to participate in the study by opt-out consent and thus 1,022 patients remained in the baseline study sample, which included 1,041 individual PCIs (as 19 patients underwent PCI twice at VNHI). There was an extremely high rate of data completeness, with only information of one lesion missed due to a lost disk (0.1%). Ninety-eight percent of fields were fully filled, with the exception of oral anti-coagulant therapy as patients had difficulty recognising the kinds of drug used and unknown information about their referral to cardiac rehabilitation. The successful follow-up rate at 30 days was high, with 993 patients followed-up (97.2%).

Of the 25 invited cardiovascular professionals, 12 consented to participate in the qualitative survey (response rate: 48%). These 12 respondents included 5 cardiologists with administrative leadership at VNHI, 3 clinical cardiologists and 4 nurses. Several key additional factors concerning the successful implementation of a PCI registry in Vietnam were raised. Nine respondents agreed with the importance of standardised data collection forms as used in the registry. Other key facilitating factors were also emphasized, including well-trained investigators, the use of professional clinical audit, and strong support from leaders of target cardiac institutions. They also raised concerns regarding the sustainability of such a study at VNHI, including lack of data storage systems, sufficient funding for infrastructure and human resources, and strong commitment from hospital leaders.

### Registry viability

The viability of the pilot PCI registry in VNHI was determined by the following elements:

- Being able to recruit a representative sample into the registry. After several amendments, the data collection strategy captured all patients undergoing PCI at VNHI four days per week (2 week days with routine practice and weekend with emergency cases only). Thus, the sample recruited into the registry could be considered to be representative of coronary patients treated with PCI at VNHI when there were not sufficient resources to collect all cases.
- Data quality. In total, we collected information on 99 data fields, and overall we had 98% data completeness. The reasons for this high completeness were the strong cooperation of patients and the availability of data resources (e.g., medical records, disks and machines in the catheterization laboratory). Additionally, the local clinical audit was performed monthly by well-trained staff, including case ascertainment (checking the collection of eligible data) and data quality assessment (reviewing source of data collection). Overall, 2% of cases were randomly checked and there were no significant errors in choosing patients and medical records. Thus, the quality and accuracy of data collected at VNHI was ensured.
- Costs for and time taken to collect the data by hospital staff. It took approximately an hour to complete a CRF, including 15 minutes for interviewing patients, abstracting data from medical records and reading procedure information in the secured disks with the cardiologist. In further studies, data collection will be the responsibility of hospital nurses. Therefore, the actual time for completing the data collection form would be around or less than one hour because of the familiarity with routine PCI practice. While hospital nurses will do data collection as part of routine clinical activity, we estimated the cost required for data collection. If we use an average monthly income of a nurse at VNHI, which is approximately 1066 USD^1^ [24], then the estimated time-cost of baseline data collection for one case, which is the income of hospital nurse in 1 hour, was equivalent to 1066/(30*8) = 4.4 USD.^2^ In follow-up survey, approximate 15–30 minutes (phone or direct interviews) will be required for each patient, which was roughly equivalent to 1.1–2.2 USD.
- The level of support from patients, clinical staff and the leader team. In the pilot registry, patients at VNHI had shown their strong engagement with the registry and there were no significant difficulties regarding the patients noted. The leader team and the hospital staff were well aware of the necessity to develop such a registry at the VNHI and in Vietnam and shown their universal support. They also belief that the establishment of the registry would be successful if there were sufficient data storing system, sufficient funding for human resources and improving the infrastructure, and strong commitment from hospital leaders.

## Discussion

In the context of the growing interest in developing clinical quality registries worldwide, this paper reports the development of the first PCI registry in Vietnam, using and adapting experiences from longstanding registries in Australia [19,25]. The work to date has demonstrated a PCI registry to be feasible and suitable for Vietnamese circumstances, providing a significant opportunity to extend the approach to other cardiac centres looking to replicate the model. Importantly, implementing such a model not only provides crucial feedback on the performance of PCI for Vietnamese clinicians and cardiac care providers but also allowing a robust comparison with other regional registries such as those involved in the ASPECT collaboration [14] as well as contributing to the literature on the use of PCI.

In comparison with other regional PCI registries in Thailand, China, India and Malaysia, the methodology in our pilot registry had some similarities, including using a standard abstraction form to collect consecutive patients undergoing PCI, providing sufficient training for investigators prior to data collection, performing clinical audit to ensure data quality and conducting follow-ups at 30 days and 12 months to investigate the outcomes of PCI [8,9,12,13]. Nonetheless, due to resource constraint, we did not approach all the patients undergoing PCI in the study period. The CRF was also completed in a paper format only and data obtained were not transferred to a web based system as other PCI registries [8,9,12]. Therefore, further studies might apply our methodology if there is limited resources or overcome our drawbacks if there is sufficient funding.

Although it is at an early stage, we are optimistic about the viability of the PCI model that we have implemented at the VNHI. The success of the PCI registry at the VNHI to date, is due to a variety of key factors such as the high number of patients undergoing PCI and their strong engagement with clinicians, the availability of data resources, and the supportive hospital staff team. We also faced several challenges in the first registry implement at VNHI, including high workload which might affect the time spent on data collection by the hospital staff and the lack of electronic record systems which made it difficult to collect comprehensive information when patients revisited the hospital. However, these obstacles can be minimised by providing sufficient training for clinical investigators, specifying sections of the data forms for investigators and conducting more detailed follow-up surveys.

One limitation is that the registry was conducted at VNHI, the leading cardiac centre in Vietnam where there is a highest patient throughput. Therefore, extending the methodology in establishing PCI registries in smaller settings with potentially less experienced staff may require a modified approach to that which we have done, but we believe this current work represents an important first step in doing so. Another potential concern may be bias in data collection, though attempt was made to enrol a representative sample. Thus, where sufficient resources are available, collecting all patients undergoing PCI in the study period would be recommended.

## Conclusion

This paper describes the methodology of establishing the first PCI registry at the leading cardiac centre in Vietnam and reports on the viability of this model. We hope that the successful implementation of a PCI registry at VNHI will encourage other cardiac intervention centres in Vietnam to adopt this model in their daily practice and by doing so, enables the opportunity to develop a nationwide PCI registry.

## Additional File

The additional file for this article can be found as follows:

10.5334/gh.782.s1 Appendix. PCI Form 1 – Baseline.

## Figures and Tables

**Table:** 

ASPECT	Asia Pacific Evaluation of Cardiovascular Therapies
BARC	Bleeding Academic Research Consortium
CHD	Coronary heart disease
CRF	Case reported form
PCI	Percutaneous coronary intervention
VCOR	Victorian Cardiac Outcomes Registry
VNHI	Vietnam National Heart Institute

## Footnotes

The exchange rate is 23.150 VND.

30 days were represented for 22 working days and extra shifts of hospital nurses.

## Author Information

**Hoa T. T. Vu**
      Hoa Thi Thanh Vu is a medical doctor by training and received her Master of Public Health in Vietnam. She currently studies as a PhD student in School of Public Health, Curtin University, Australia, and is a lecturer in Department of Medical Community at Thai Nguyen University of Medicine and Pharmacy, Vietnam. She is interested in chronic disease epidemiology and prevention. Her current research focus on establishing a percutaneous coronary intervention registry in Vietnam.
**Hoai T. T. Nguyen**
      Doctor Hoai Nguyen Thi Thu is the Vice Director of the Vietnam National Heart Institute. She received an M.D. degree and a PhD in cardiology from Hanoi Medical University, Vietnam. She also undertook post-graduate training and fellowship at Monash University, Australia during 2006–2007 and in France in 2011, respectively. In addition, Dr Nguyen is a lecturer in cardiology at Hanoi Medical University and the Deputy Head of Department of Internal Medicine at School of Medicine and Pharmacy, Vietnam National University. She also is the Secretary General of Vietnamese Society of Echocardiography. Her interests includes preventive cardiology, clinical cardiology and echocardiography.
**Hung M. Pham**
      A/Professor HUNG MANH PHAM is the Director of The Vietnam National Heart Institute. He received an M.D. degree and a PhD in cardiology from Hanoi Medical University (HMU), Vietnam. He also undertook the fellowship of Interventional Cardiology in South Korea during 1998–1999 and in USA in 2000 and 2002, respectively. Additionally, he is the current head of department of Cardiology, HMU and vice editor in Chief, Journal of Vietnamese Cardiology. He is interested in a variety area of cardiology, such as mitral balloon valvuloplasty, congenital heart disease intervention, coronary intervention and stem cell.
**Loi D. Do**
      **Biography/Background**
  Professor Loi DO DOAN MD, PhD, FACC, Former Chair of Cardiology Deparment at Hanoi Medical University and Director of Vietnam National Heart Institute. He is currently serving as a Vice President of the Vietnam Heart Association. He was a Resident in Hospital of Paris VI University 1988–1990, visiting reseach scholar at Stanford University 1998–1999.
  **Qualifications**
  Doctor of Philosophy 2002
  Medical Doctor 1982 (Rostov Medical School – USSR)
  **Research Interests**
  His research interests include clinical Cardiology, Echocardiography and Arterial Hypertension.
**Quang N. Nguyen**
      Quang Ngoc Nguyen is Vice Director of Department of Cardiology, Hanoi Medical University and Head of Coronary Care Unit, Vietnam National Heart Institute, Hanoi, Vietnam. His research interests focus on interventional cardiology (transcatheter coronary and cardiac structural intervention) and cardiovascular disease epidemiology (CVD risk factors trend/burden and interventions in community).
**Richard Norman**
      Richard Norman is an Associate Professor in Health Economics, based at the Curtin University School of Public Health. His research focuses on economic evaluation of healthcare, on the measurement and valuation of quality of life, and on patient and population preferences for different aspects of healthcare.
**Rachel R. Huxley**
      Professor Rachel Huxley is Associate Pro-Vice Chancellor (Research) for the College of Science, Health and Engineering, and also Co-Director of the recently established Research Centre for Cardiovascular Biology and Disease at La Trobe University. Previously, she was the Head of School of Public Health at Curtin University, Australia. She completed her undergraduate, PhD and post- doctoral training in Oxford University. She has published more than 200 research articles, has a Google Scholar H-index of 64 and >20,000 citations. She currently holds several competitive research grants as Principal Investigator in areas related to obesity, diabetes and women’s health.
**Ngoc M. Pham**
      Ngoc Minh Pham is a medical doctor by training from Vietnam and received his Master of Public Health and PhD in Medical Science from the University of Melbourne in Australia and Kyushu University in Japan, respectively. He currently works as a visiting research associate at Curtin University, Australia and a lecturer in epidemiology at Thai Nguyen University of Medicine and Pharmacy, Vietnam. He has eight years of postdoctoral research experience in the epidemiology and prevention of lifestyle-related diseases and disorders, particularly type 2 diabetes, metabolic syndrome and cardiovascular risk factors.
**Crystal M. Y. Lee**
      Crystal Lee is a Senior Research Fellow at the School of Psychology and Public Health, La Trobe University and Honorary Research Fellow at the Boden Institute of Obesity, Nutrition, Exercise & Eating Disorders, University of Sydney. Her research interests focus on the use of large epidemiological datasets and published data to study obesity, diabetes, and cardiovascular disease in adults. Her current projects include the analysis of primary care and registry datasets in Australia.
**Christopher M. Reid**
      Professor Christopher Reid is a John Curtin Distinguished Professor and Associate Director of the Monash Centre for Cardiovascular Research and Education in Therapeutics. He is a cardiovascular epidemiologist and clinical trialist with a specific interest in quality improvement and outcomes research. He holds a NHMRC Senior Research Fellowship and has had continual NHMRC research funding since 1997. He has published more than 250 papers with over 6785 citations (Google Scholar H-index 41). Over the past 5 years (from 2009), he has received 17 peer reviewed grants as a Chief Investigator from National and International funding agencies totalling over $70M.
