# Supplementary material for: Establishment of a Percutaneous Coronary Intervention Registry in Vietnam: Rationale and Methodology
Source: Glob Heart. 2020 Apr 8;15(1):30. doi: 10.5334/gh.782 (PMC7218793; doi:10.5334/gh.782)
Supplement: Appendix. — PCI Form 1 – Baseline. [file gh-15-1-782-s1.pdf]

5282451658

Patient ID | | | |

Curtin University

CCRE Centre of Clinical Research and Education

VIỆN TIM MẠCH VIỆT NAM

PCI Form 1 - Baseline

Patient Details

1.1.1 Medical record number

1.1.2 Surname

1.1.3 First name

1.1.4 Middle name

1.1.5 Gender ◯ Male ◯ Female

1.1.6 Date of Birth d d / m m / y y y y

1.1.7 ID number OR ◯ Patient has no ID number

1.1.8 Province code OR ◯ Patient has no province code

1.1.9 Primary Phone No.

1.1.10 Alternate Phone No. OR ◯ Patient has no alternate phone No

1.1.11 Ethnic group OR ◯ Patient has no ethnic code

1.1.12 Poor status ◯ Poor ◯ Near poor ◯ Other

1.1.13 Educational level ◯ Primary school ◯ Secondary school ◯ High school ◯ Higher

1.1.14 Occupation ◯ Official worker ◯ Manual worker ◯ Farmer ◯ Others

1.1.15 Monthly income/ person VND

Admission Data

2.1.1 Admission date at PCI hospital d d / m m / y y y y

2.1.2 Time of arrival at PCI hospital h h : m m

2.1.3 PCI Procedure date d d / m m / y y y y

2.1.4 PCI Procedure time h h : m m

2.1.5 Patient height cm

2.1.6 Patient weight kg

Clinical Symptoms

2.2.1 Acute coronary syndrome (ACS) ○ NO ○ YES → **if NO** *proceed to Clinical Presentation* ***2.3.1***

2.2.2 Date of ACS symptom onset (ACS onset must be <7 days ago) d d / m m / y y y y Accuracy Code

2.2.3a Time of ACS symptom onset h h : m m

2.2.3b Time estimated? ○ NO ○ YES ***OR*** ○ Onset time not available

2.2.4 Type of ACS ○ Unstable angina ○ NSTEMI ○ STEMI

For STEMI Patients ONLY:

2.2.5 Inter hospital transfer ○ NO ○ YES

2.2.6 Pre hospital notification ○ NO ○ YES

2.2.7 Balloon / device time h h : m m

Clinical Presentation

2.3.1 Cardiogenic shock ○ NO ○ YES

2.3.2 Out of hospital cardiac arrest ○ NO ○ YES

2.3.3 In-hospital pre-procedure cardiac ○ NO ○ YES

2.3.4 In-hospital pre-procedural intubation ○ NO ○ YES

Pre-Procedural LV Function

3.1.1a LVEF test performed ○ NO ○ YES → **if YES** *complete 3.1.1b - 3.1.3*

3.1.1b Date of most recent LVEF test d d / m m / y y y y Accuracy Code

3.1.2 Most recent LVEF test type ○ Echocardiography ○ Gated cardiac blood pool scan ○ Myocardial perfusion scan ○ Angiography ○ Magnetic resonance imaging (MRI) ○ Not stated/inadequately described

3.1.3 Most recent ejection fraction: Digitally derived % ***OR*** Estimated ○ Normal (> 50%) ○ Moderate (35 - 44%) ○ Mild (45 - 49%) ○ Severe (< 35%)

Pre-Procedural Risk Factors

3.2.1a Diabetes medication ○ NO ○ YES → ***if YES*** 3.2.1b Medication type ○ Oral ○ Insulin

3.2.2 Hypertension medication ○ NO ○ YES

3.2.3 Dyslipidemia medication ○ NO ○ YES

3.2.4 Peripheral vascular disease history ○ NO ○ YES

3.2.5 Cerebrovascular disease history ○ NO ○ YES

3.2.6 Chronic oral anti-coagulant therapy ○ NO ○ YES

3.2.7a Previous CABG ○ NO ○ YES → **if YES** 3.2.7b Date most recent CABG d d / m m / y y y y Accuracy Code

3.2.8a Previous PCI ○ NO ○ YES → **if YES** 3.2.8b Date of most recent PCI d d / m m / y y y y Accuracy Code

Patient ID ☐☐☐☐

Pre-Procedural Renal Status

3.3.1a Last pre-procedure creatinine ☐☐☐☐ μmol/L **OR** ○ Creatinine results not available

3.3.1b Date of test
cannot be >60days prior to PCI
☐☐ / ☐☐ / ☐☐☐☐ (d d / m m / y y y y) ☐☐☐ Accuracy Code

3.3.2 Dialysis therapy (chronic renal failure) ○ NO ○ YES **if NO** → 3.3.4

3.3.3 Functioning renal transplant ○ NO ○ YES

3.3.4 Acute renal replacement therapy ○ NO ○ YES

Peri-procedural Medication

3.4.1a Fibrinolytic therapy ○ NO ○ YES **if YES** → 3.4.1b

3.4.1b Fibronolytic therapy <= 24 ○ NO ○ YES

3.4.2 Medications given <= 24 hours prior to and during the PCI procedure (Check ALL that apply)

☐ Glycoprotein IIb/IIIa inhibitor therapy ☐ Antithrombin therapy ☐ Ticagrelor ☐ Clopidogrel/Ticlopidine ☐ Aspirin

Procedure Details

4.1.1 PCI indication - select ONE *(PCI Indication must not contradict ACS presentation coding on page 1)*

- ○ Primary PCI for STEMI < 12hrs
- ○ PCI for STEMI >12hr (unstable)
- ○ PCI for STEMI >12hr (stable)
- ○ STEMI (stable after full-dose thrombolytics)
- ○ STEMI (unstable after full-dose thrombolytics) non-rescue PCI
- ○ Rescue PCI for STEMI (failed thrombolytics)
- ○ PCI post cardiac arrest/cardiogenic shock (non MI)
- ○ PCI for NSTEMI
- ○ PCI for unstable angina
- ○ PCI for ACS >7days ago (stable)
- ○ PCI for stable angina
- ○ Staged PCI
- ○ No angina / angina equivalent
- ○ Other (give reason) ____________________

4.1.2 Reason for PCI (non-acute / non-ACS patients)
(Answer if PCI indication is not coded as acute STEMI, NSTEMI, Unstable Angina or Recent ACS)

- ○ High Grade Stenosis (>70%)
- ○ Medium Grade Stenosis (50-70%)
- ○ Low Grade Stenosis (< 50%)
- ○ Function test positive (+)
- ○ Functional test negative (-)
- ○ Functional test equivocal (?)
- ○ Functional test not done (x)

4.2.1 Percutaneous entry location ○ Brachial ○ Radial ○ Femoral

4.2.2a Adjunctive device required ○ NO ○ YES **if YES** → 4.2.2b

4.2.2b Type of adjunctive device(s) (check ALL that apply)

- ☐ Intravascular Ultrasound
- ☐ Optical coherence tomography
- ☐ Thrombus aspiration device
- ☐ Distal or proximal protection device
- ☐ Rotational arthrectomy
- ☐ Fractional flow reserve
- ☐ Other (specify) ____________________

4.2.3 Procedural intubation required ○ NO ○ YES

4.2.4a Mechanical ventricular support required ○ NO ○ YES **if YES** → 4.2.4b

4.2.4b Type of mechanical support required ○ IABP ○ ECMO ○ LVAD

0560451652

Patient ID | | | | |

Lesion 1 - Procedure Details (Lesion & Device Data)

The following questions are to be answered for lesion 1 ONLY.

4.3.1 Lesion Location Code [ ][ ][ ] *Refer to Lesion Location Map*

4.3.2 Lesion Type ◯ A ◯ B1 ◯ B2 ◯ C

4.3.3 Chronic total occlusion (CTO) ◯ NO ◯ YES

4.3.4a In-stent restenosis (ISR) ◯ NO ◯ YES → **if YES** 4.3.4b Stent thrombosis ◯ NO ◯ YES

4.3.5 Lesion successfully treated ◯ NO ◯ YES

4.4.1a Total number of stents (current lesion) [ ] **if = 0** → 4.4.2; **if > 0** *continue to 4.4.1 b & c*

4.4.1b Total length of stents (current lesion) [ ][ ][ ] mm

4.4.1c Type of stent(s) implanted

◯ Bare metal stents (BMS) ◯ BVS (non-drug scaffold)

◯ Drug-eluting stents (DES) ◯ Drug eluting BVS (DE-BVS)

◯ Mixed stents (BMS & DES) ◯ Other (specify) ______________________

4.4.2 Type of balloon(s) deployed

◯ No balloon / stent used

◯ Plain balloon

◯ Drug-eluting balloon

Lesion 2 - Procedure Details (Lesion & Device Data)

The following questions are to be answered for lesion 2 ONLY.

4.3.1 Lesion Location Code [ ][ ][ ] *Refer to Lesion Location Map*

4.3.2 Lesion Type ◯ A ◯ B1 ◯ B2 ◯ C

4.3.3 Chronic total occlusion (CTO) ◯ NO ◯ YES

4.3.4a In-stent restenosis (ISR) ◯ NO ◯ YES → **if YES** 4.3.4b Stent thrombosis ◯ NO ◯ YES

4.3.5 Lesion successfully treated ◯ NO ◯ YES

4.4.1a Total number of stents (current lesion) [ ] **if = 0** → 4.4.2; **if > 0** *continue to 4.4.1 b & c*

4.4.1b Total length of stents (current lesion) [ ][ ][ ] mm

4.4.1c Type of stent(s) implanted

◯ Bare metal stents (BMS) ◯ BVS (non-drug scaffold)

◯ Drug-eluting stents (DES) ◯ Drug eluting BVS (DE-BVS)

◯ Mixed stents (BMS & DES) ◯ Other (specify) ______________________

4.4.2 Type of balloon(s) deployed

◯ No balloon / stent used

◯ Plain balloon

◯ Drug-eluting balloon

Lesion 3 - Procedure Details (Lesion & Device Data)

The following questions are to be answered for lesion 3 ONLY.

4.3.1 Lesion Location Code [ ][ ][ ] *Refer to Lesion Location Map*

4.3.2 Lesion Type ◯ A ◯ B1 ◯ B2 ◯ C

4.3.3 Chronic total occlusion (CTO) ◯ NO ◯ YES

4.3.4a In-stent restenosis (ISR) ◯ NO ◯ YES → **if YES** 4.3.4b Stent thrombosis ◯ NO ◯ YES

4.3.5 Lesion successfully treated ◯ NO ◯ YES

4.4.1a Total number of stents (current lesion) [ ] **if = 0** → 4.4.2; **if > 0** *continue to 4.4.1 b & c*

4.4.1b Total length of stents (current lesion) [ ][ ][ ] mm

4.4.1c Type of stent(s) implanted

◯ Bare metal stents (BMS) ◯ BVS (non-drug scaffold)

◯ Drug-eluting stents (DES) ◯ Drug eluting BVS (DE-BVS)

◯ Mixed stents (BMS & DES) ◯ Other (specify) ______________________

4.4.2 Type of balloon(s) deployed

◯ No balloon / stent used

◯ Plain balloon

◯ Drug-eluting balloon

Lesion Location Map

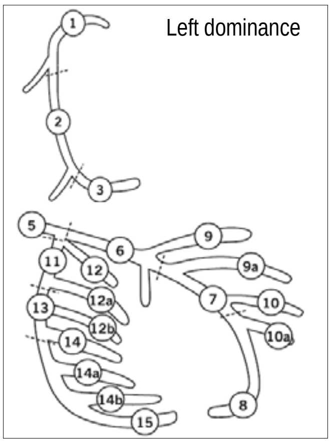

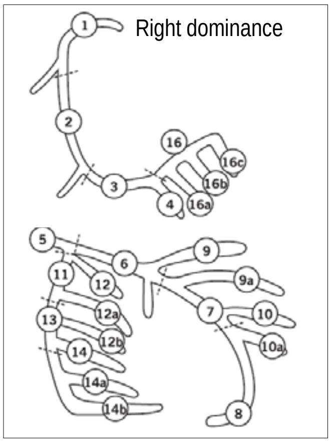

Coronary Artery Segment Codes

1 RCA proximal
2 RCA mid
3 RCA distal
4 PDA
5 Left main
6 LAD proximal
7 LAD mid
8 LAD apical (distal)
9 D1 First diagonal
9a D1a First diagonal a
10 D2 Second Diagonal
10a D2a Second Diagonal a
11 Proximal circumflex
12 Intermediate / anterolateral
12a Obtuse marginal a
12b Obtuse marginal b
13 Distal cirumflex
14 Left posterolateral
14a Left posterolateral a
14b Left posterolateral b
15 Posterior descending
16 Posterolateral from RCA
16a Posterolateral from RCA a
16b Posterolateral from RCA b
16c Posterolateral from RCA c
17 Internal mammary graft
18 Radial artery graft
19 Saphenous vein graft

Note: Where serial stenoses are treated with overlapping stents / devices, classify as ONE lesion.

Lesion 4 - Procedure Details (Lesion & Device Data)

The following questions are to be answered for lesion 4 ONLY.

4.3.1 Lesion Location Code _Refer to Lesion Location Map_

4.3.2 Lesion Type ○ A ○ B1 ○ B2 ○ C

4.3.3 Chronic total occlusion (CTO) ○ NO ○ YES

4.3.4a In-stent restenosis (ISR) ○ NO ○ YES → **if YES** 4.3.4b Stent thrombosis ○ NO ○ YES

4.3.5 Lesion successfully treated ○ NO ○ YES

4.4.1a Total number of stents (current lesion) **if = 0** → 4.4.2 Type of balloon(s) deployed ○ No balloon / stent used ○ Plain balloon ○ Drug-eluting balloon

if > 0 _continue to 4.4.1 b & c_

4.4.1b Total length of stents (current lesion) mm

4.4.1c Type of stent(s) implanted ○ Bare metal stents (BMS) ○ BVS (non-drug scaffold) ○ Drug-eluting stents (DES) ○ Drug eluting BVS (DE-BVS) ○ Mixed stents (BMS & DES) ○ Other (specify) ____

Lesion 5 - Procedure Details (Lesion & Device Data)

The following questions are to be answered for lesion 5 ONLY.

4.3.1 Lesion Location Code _Refer to Lesion Location Map_

4.3.2 Lesion Type ○ A ○ B1 ○ B2 ○ C

4.3.3 Chronic total occlusion (CTO) ○ NO ○ YES

4.3.4a In-stent restenosis (ISR) ○ NO ○ YES → **if YES** 4.3.4b Stent thrombosis ○ NO ○ YES

4.3.5 Lesion successfully treated ○ NO ○ YES

4.4.1a Total number of stents (current lesion) **if = 0** → 4.4.2 Type of balloon(s) deployed ○ No balloon / stent used ○ Plain balloon ○ Drug-eluting balloon

if > 0 _continue to 4.4.1 b & c_

4.4.1b Total length of stents (current lesion) mm

4.4.1c Type of stent(s) implanted ○ Bare metal stents (BMS) ○ BVS (non-drug scaffold) ○ Drug-eluting stents (DES) ○ Drug eluting BVS (DE-BVS) ○ Mixed stents (BMS & DES) ○ Other (specify) ____

Post-Procedural Cardiac Biomarkers

5.1.1 Post-procedural cardiac biomarker levels measured within 24 hours ○ NO ○ YES

↳ **if NO** _go straight to **5.3.1**_

5.2.1a Peak Troponin <= 24 hours [ ][ ][ ][ ] . [ ][ ] ○ Tn-I ng/L ○ Tn-T ng/L **OR** ○ Not measured

5.2.1b Date and time of Troponin levels measured d d / m m / y y y y h h : m m

5.2.2a Peak CK-MB <= 24 hours (U/L) U/L **OR** ○ Not measured

5.2.2b Date and time of CK-MB levels measured d d / m m / y y y y h h : m m

5.2.3a Peak CK <= 24hours (U/L) U/L **OR** ○ Not measured

5.2.3b Date and time of CK levels measured d d / m m / y y y y h h : m m

In-Hospital Complications

5.3.1 New renal impairment ○ NO ○ YES **OR** ○ Post-procedural serum creatinine not measured
Creatinine rise ≥ 44.2 µmol/L OR ≥25% (up to 5 days following index PCI)

5.3.2 New requirement for dialysis ○ NO ○ YES

5.3.3 In-hospital cardiogenic shock ○ NO ○ YES

5.3.4 In-hospital new or recurrent MI ○ NO ○ YES

5.3.5a In hospital PCI ○ NO ○ YES →
(Subsequent in-hospital angioplasty after index PCI)

5.3.5b Planned in-hospital PCI ○ NO ○ YES

5.3.5c(i) In-hospital TVR (PCI) ○ NO ○ YES

5.3.5c(ii) In-hospital target LESION revascularisation ○ NO ○ YES

Please record any subsequent PCi as a new event

5.3.6a In hospital cardiothoracic surgery ○ NO ○ YES →
(Subsequent cardiothoracic surgery
Note: Includes acute transfers to tertiary referral centres)

5.3.6b Planned cardiothoracic surgery ○ NO ○ YES

5.3.6c In-hospital TVR (CABG) ○ NO ○ YES

5.3.7 In-hospital stent thrombosis ○ None ○ Definite ○ Probable ○ Possible

5.3.8 In-hospital bleeding
(Please refer to BARC definitions)
○ Type 0 ○ Type 3a ○ Type 4
○ Type 1 ○ Type 3b ○ Type 5a
○ Type 2 ○ Type 3c ○ Type 5b

5.3.9a In-hospital stroke ○ NO ○ YES → 5.3.9b Stroke type ○ Haemorrhagic ○ Ischaemic

Discharge Details

6.1.1a Discharge Status
○ Home ○ Local or referring hospital
○ Hospital in the Home ○ Tertiary referral centre
○ Rehabilitation Unit/Hospital ○ Hospital mortality

6.1.1b Date of discharge / Death __ __ / __ __ / __ __ __ __
d d m m y y y y

6.1.2 Cardiac rehabilitation referral ○ NO ○ YES ○ UNKNOWN

Discharge Medications

6.2.1a	Aspirin	○ NO	○ YES	○ Contraindicated	○ Not collected
6.2.1b	Clopidogrel/ Ticlopidine	○ NO	○ YES	○ Contraindicated	○ Not collected
6.2.1c	Ticagrelor	○ NO	○ YES	○ Contraindicated	○ Not collected
6.2.1d	Beta Blockers	○ NO	○ YES	○ Contraindicated	○ Not collected
6.2.1e	ACE/ARB	○ NO	○ YES	○ Contraindicated	○ Not collected
6.2.1f	Statin	○ NO	○ YES	○ Contraindicated	○ Not collected
6.2.1g	Other Lipid Lowering Therapies	○ NO	○ YES	○ Contraindicated	○ Not collected
6.2.1h	Oral anticoagulation therapies	○ NO	○ YES	○ Contraindicated	○ Not collected

PCI Form 2 - 30 days Follow-Up

Patient ID ☐☐☐☐

Patient Details

1.1.1 Medical record number ___________

1.1.7 ID number ☐☐☐☐☐☐☐☐☐☐☐☐

1.1.2 Surname ☐☐☐☐☐☐☐☐

1.1.3 First name ☐☐☐☐☐☐☐☐

1.1.4 Gender ○ Male ○ Female

1.1.6 Date of birth ☐☐ / ☐☐ / ☐☐☐☐ (d d / m m / y y y y)

30 Day Outcomes

7.1.1 Date of follow-up ☐☐ / ☐☐ / ☐☐☐☐ (d d / m m / y y y y)

7.1.2a Follow-up status (30 days) ○ Alive ○ Deceased ○ Unknown → If patient lost to follow-up *do not continue*

If deceased *answer 7.1.2b-c and continue through to 7.1.8*

7.1.2b Date of death ☐☐ / ☐☐ / ☐☐☐☐ (d d / m m / y y y y) ☐☐☐ Accuracy Code

7.1.2c Primary cause of death ○ Cardiac ○ Non - cardiac ○ Uncertain

7.1.3 New heart failure ○ NO ○ YES ○ UNKNOWN

7.1.4 New myocardial infarction ○ NO ○ YES ○ UNKNOWN

7.1.5 New stent-thrombosis ○ None ○ Definite ○ Probable ○ Possible ○ UNKNOWN

7.1.6 New bleeding event (Please refer to the BARC definition) ○ Type 0 ○ Type 1 ○ Type 2 ○ Type 3a ○ Type 3b ○ Type 3c ○ Type 4 ○ Type 5a ○ Type 5b ○ UNKNOWN

7.1.7a New stroke ○ NO ○ YES → If yes 7.1.7b Stroke type ○ Haemorrhagic ○ Ischaemic ○ UNKNOWN

7.1.8a Rehospitalisation ○ NO ○ YES → If yes *answer Questions 7.1.8b-e in Rehospitalisation Table (for each rehospitalisation up to 6 rehospitalisations)* ○ UNKNOWN → If UNK *continue to medications*

30 Day Outcomes (continued...)

Please answer for each rehospitalisation (up to 6 rehospitalisations)	Rehospitalisation 1	2	3	4	5	6
7.1.8b Cardiac rehospitalisation — If no *do not continue with items 7.1.8 c-e*	○ No ○ Yes	○ No ○ Yes	○ No ○ Yes	○ No ○ Yes	○ No ○ Yes	○ No ○ Yes
	if NO *do not continue with items 7.1.8 c-e*					
7.1.8c Planned cardiac rehospitalisation	○ No ○ Yes	○ No ○ Yes	○ No ○ Yes	○ No ○ Yes	○ No ○ Yes	○ No ○ Yes
7.1.8d (i) PCI rehospitalisation — **If no** *continue to 7.1.8e*	○ No ○ Yes	○ No ○ Yes	○ No ○ Yes	○ No ○ Yes	○ No ○ Yes	○ No ○ Yes
7.1.8d (ii) Target vessel revascularisation (PCI)	○ No ○ Yes	○ No ○ Yes	○ No ○ Yes	○ No ○ Yes	○ No ○ Yes	○ No ○ Yes
7.1.8d (iii) Target lesion revascularisation (TLR)	○ No ○ Yes	○ No ○ Yes	○ No ○ Yes	○ No ○ Yes	○ No ○ Yes	○ No ○ Yes
7.1.8e (i) CABG rehospitalisation — **If no** *continue to medications*	○ No ○ Yes	○ No ○ Yes	○ No ○ Yes	○ No ○ Yes	○ No ○ Yes	○ No ○ Yes
7.1.8e (ii) Target vessel CABG	○ No ○ Yes	○ No ○ Yes	○ No ○ Yes	○ No ○ Yes	○ No ○ Yes	○ No ○ Yes

Do not complete sections 7.2 and 7.3 if patient is deceased

Medications at 30 days

7.2.1a Aspirin ○ NO ○ YES ○ Not asked

7.2.1b Clopidogrel/Ticlopidine ○ NO ○ YES ○ Not asked

7.2.1c Ticagrelor ○ NO ○ YES ○ Not asked

Quality of Life (QoL) at 30 days

○ All QoL not asked

7.3.1a Mobility ○ No problem ○ Some problem ○ Confined to bed ○ Not asked

7.3.1b Personal care ○ No problem ○ Some problem ○ Unable to wash/ dress ○ Not asked

7.3.1c Usual activities ○ No problem ○ Some problem ○ Unable to perform usual activities ○ Not asked

7.3.1d Pain/discomfort ○ No problem ○ Some problem ○ Extreme pain/ discomfort ○ Not asked

7.3.1e Anxiety/depression ○ No problem ○ Some problem ○ Extreme anxiety/ depression ○ Not asked

7.3.2 Own health state today ☐☐☐ ○ Not asked

0 = worst; 100 = best

PCI Follow up Telephone Script (EQ-5D Quality of life)

Curtin University | CCRE Centre of Clinical Research and Education | VIỆN TIM MẠCH VIỆT NAM

Quality of life

It is suggested that the telephone administrator follows the script of the EQ-5D. Although allowance should be made for the interviewer's particular style of speaking, the wording should be followed as closely as possible. **In the case of the EQ-5D descriptive system, the precise wording must be followed**.

It is recommended that the administrator has a copy of the EQ-5D in front of them as it is administered over the telephone.
If the respondent has difficulty with regard to which box to tick, the administrator should repeat the question verbatim and ask the respondent to answer in a way that most closely resembles their thoughts about their health state today.

EQ-5D

"We are trying to find out what you think about your health. I will first ask you a few brief and simple questions about your own health state today. I will then ask you to do a rather different task that involves rating your health on a measuring scale. I will explain the tasks fully as I go along but please interrupt me if you do not understand something or if things are not clear to you. Please remember that there are no right or wrong answers. We are interested here only in your personal view."

"First I am going to read out some questions. Each question has a choice of three answers. Please tell me which answer best describes your own health state today. Do not choose more than one answer in each group of questions."

1. In terms of mobility, would you say you have
- No problem walking around
- Some problems walking around
- Are you confined to bed

2. In term of personal care (washing, dressing), would you say you are
- No problems with personal care
- Some problems washing and dressing myself
- Unable to wash/dress myself

3. In terms of usual activities (e.g. work, study,housework, family of leisure activities), would you say you have
- No problem performing my usual activities
- Some problem performing my usual activies
- Unable to perform my usual activities

4. In terms of pain/ discomfort, would you say you have
- No pain/ discomfort
- Moderate pain/ discomport
- Extreme pain/ discomfort

5. Interm of anxiety/ depression, would you say you are
- Not anxious/depressed
- Moderately anxious/depressed
- Extremely anxious/depressed

6."I would like to ask you to do a rather different task. To help you say how good or bad your health state is, I'd like you to try to picture in your mind a scale that looks a bit like a thermometer. The best health state you can imagine is marked 100 at the top of the scale and the worst state you can imagine is marked zero at the bottom.
Please tell me the point on this scale where you would put your own health state today."

Please indicate numerically, on a scale between 0-100, where the patient visualises theri own health state TODAY.

Record the value as per the patients's answer. Remember, "100" is as good as the interviewee has ever felt in their life.

PCI Form 2 - 12 months Follow-Up

Patient ID ☐☐☐☐

Patient Details

1.1.1 Medical record number |_|_|_|_|_|_|_|_|_|_|_|

1.1.7 ID number ☐☐☐☐☐☐☐☐☐☐☐☐

1.1.2 Surname ☐☐☐☐☐☐☐☐

1.1.3 First name ☐☐☐☐☐☐☐☐

1.1.4 Gender ○ Male ○ Female

1.1.6 Date of birth ☐☐ / ☐☐ / ☐☐☐☐ (d d / m m / y y y y)

12 months Outcomes

7.1.1 Date of follow-up ☐☐ / ☐☐ / ☐☐☐☐ (d d / m m / y y y y)

7.1.2a Follow-up status (12 months) ○ Alive ○ Deceased ○ Unknown → If patient lost to follow-up *do not continue*

If deceased *answer 7.1.2b-c and continue through to 7.1.8*

7.1.2b Date of death ☐☐ / ☐☐ / ☐☐☐☐ (d d / m m / y y y y) ☐☐☐ Accuracy Code

7.1.2c Primary cause of death ○ Cardiac ○ Non - cardiac ○ Uncertain

7.1.3 New heart failure ○ NO ○ YES ○ UNKNOWN

7.1.4 New myocardial infarction ○ NO ○ YES ○ UNKNOWN

7.1.5 New stent-thrombosis ○ None ○ Definite ○ Probable ○ Possible ○ UNKNOWN

7.1.6 New bleeding event (Please refer to the BARC definition) ○ Type 0 ○ Type 1 ○ Type 2 ○ Type 3a ○ Type 3b ○ Type 3c ○ Type 4 ○ Type 5a ○ Type 5b ○ UNKNOWN

7.1.7a New stroke ○ NO ○ YES → If yes 7.1.7b Stroke type ○ Haemorrhagic ○ Ischaemic ○ UNKNOWN

7.1.8a Rehospitalisation ○ NO ○ YES → If yes *answer Questions 7.1.8b-e in Rehospitalisation Table (for each rehospitalisation up to 6 rehospitalisations)* ○ UNKNOWN → If UNK *continue to medications*

12 months Outcomes (continued...)

Please answer for each rehospitalisation (up to 6 rehospitalisations)	Rehospitalisation 1	2	3	4	5	6
7.1.8b Cardiac rehospitalisation<br>If no *do not continue with items 7.1.8 c-e*	○ No ○ Yes	○ No ○ Yes	○ No ○ Yes	○ No ○ Yes	○ No ○ Yes	○ No ○ Yes
	if NO *do not continue with items 7.1.8 c-e*					
7.1.8c Planned cardiac rehospitalisation	○ No ○ Yes	○ No ○ Yes	○ No ○ Yes	○ No ○ Yes	○ No ○ Yes	○ No ○ Yes
7.1.8d (i) PCI rehospitalisation<br>**If no** *continue to 7.1.8e*	○ No ○ Yes	○ No ○ Yes	○ No ○ Yes	○ No ○ Yes	○ No ○ Yes	○ No ○ Yes
7.1.8d (ii) Target vessel revascularisation (PCI)	○ No ○ Yes	○ No ○ Yes	○ No ○ Yes	○ No ○ Yes	○ No ○ Yes	○ No ○ Yes
7.1.8d (iii) Target lesion revascularisation (TLR)	○ No ○ Yes	○ No ○ Yes	○ No ○ Yes	○ No ○ Yes	○ No ○ Yes	○ No ○ Yes
7.1.8e (i) CABG rehospitalisation<br>**If no** *continue to medications*	○ No ○ Yes	○ No ○ Yes	○ No ○ Yes	○ No ○ Yes	○ No ○ Yes	○ No ○ Yes
7.1.8e (ii) Target vessel CABG	○ No ○ Yes	○ No ○ Yes	○ No ○ Yes	○ No ○ Yes	○ No ○ Yes	○ No ○ Yes

Do not complete sections 7.2 and 7.3 if patient is deceased

Medications at 12 months

7.2.1a Aspirin ○ NO ○ YES ○ Not asked

7.2.1b Clopidogrel/Ticlopidine ○ NO ○ YES ○ Not asked

7.2.1c Ticagrelor ○ NO ○ YES ○ Not asked

Quality of Life (QoL) at 12 months

○ All QoL not asked

7.3.1a Mobility ○ No problem ○ Some problem ○ Confined to bed ○ Not asked

7.3.1b Personal care ○ No problem ○ Some problem ○ Unable to wash/ dress ○ Not asked

7.3.1c Usual activities ○ No problem ○ Some problem ○ Unable to perform usual activities ○ Not asked

7.3.1d Pain/discomfort ○ No problem ○ Some problem ○ Extreme pain/ discomfort ○ Not asked

7.3.1e Anxiety/depression ○ No problem ○ Some problem ○ Extreme anxiety/ depression ○ Not asked

7.3.2 Own health state today ☐☐☐ ○ Not asked

0 = worst; 100 = best

Version 1.4 October 2015

PCI Follow up Telephone Script (EQ-5D Quality of life)

Curtin University | CCRE Centre of Clinical Research and Education | VIỆN TIM MẠCH VIỆT NAM

Quality of life

It is suggested that the telephone administrator follows the script of the EQ-5D. Although allowance should be made for the interviewer's particular style of speaking, the wording should be followed as closely as possible. **In the case of the EQ-5D descriptive system, the precise wording must be followed**.

It is recommended that the administrator has a copy of the EQ-5D in front of them as it is administered over the telephone.
If the respondent has difficulty with regard to which box to tick, the administrator should repeat the question verbatim and ask the respondent to answer in a way that most closely resembles their thoughts about their health state today.

EQ-5D

"We are trying to find out what you think about your health. I will first ask you a few brief and simple questions about your own health state today. I will then ask you to do a rather different task that involves rating your health on a measuring scale. I will explain the tasks fully as I go along but please interrupt me if you do not understand something or if things are not clear to you. Please remember that there are no right or wrong answers. We are interested here only in your personal view."

"First I am going to read out some questions. Each question has a choice of three answers. Please tell me which answer best describes your own health state today. Do not choose more than one answer in each group of questions."

1. In terms of mobility, would you say you have

- No problem walking around
- Some problems walking around
- Are you confined to bed

2. In term of personal care (washing, dressing), would you say you are

- No problems with personal care
- Some problems washing and dressing myself
- Unable to wash/dress myself

3. In terms of usual activities (e.g. work, study,housework, family of leisure activities), would you say you have

- No problem performing my usual activities
- Some problem performing my usual activies
- Unable to perform my usual activities

4. In terms of pain/ discomfort, would you say you have

- No pain/ discomfort
- Moderate pain/ discomport
- Extreme pain/ discomfort

5. Interm of anxiety/ depression, would you say you are

- Not anxious/depressed
- Moderately anxious/depressed
- Extremely anxious/depressed

6."I would like to ask you to do a rather different task. To help you say how good or bad your health state is, I'd like you to try to picture in your mind a scale that looks a bit like a thermometer. The best health state you can imagine is marked 100 at the top of the scale and the worst state you can imagine is marked zero at the bottom.
Please tell me the point on this scale where you would put your own health state today."

Please indicate numerically, on a scale between 0-100, where the patient visualises theri own health state TODAY.

Record the value as per the patients's answer. Remember, "100" is as good as the interviewee has ever felt in their life.
